# Variation in Phenolics, Flavanoids, Antioxidant and Tyrosinase Inhibitory Activity of Peach Blossoms at Different Developmental Stages

**DOI:** 10.3390/molecules201119709

**Published:** 2015-11-18

**Authors:** Jie-Chao Liu, Zhong-Gao Jiao, Wen-Bo Yang, Chun-Ling Zhang, Hui Liu, Zhen-Zhen Lv

**Affiliations:** Zhengzhou Fruit Research Institute, Chinese Academy of Agricultural Sciences, Zhengzhou 450009, China; liujiechao@caas.cn (J.-C.L.); yangwenbo@caas.cn (W.-B.Y.); zhangchunling@caas.cn (C.-L.Z.); liuhui@caas.cn (H.L.); lvzhenzhen@caas.cn (Z.-Z.L.)

**Keywords:** peach, blossom, phenolic, flavanoid, antioxidant, tyrosinase, development

## Abstract

Peach blossoms were harvested and classified into six developmental stages: (I) bud emerging stage; (II) middle bud stage; (III) large bud stage; (IV) initial-flowering stage; (V) full-flowering stage; and (VI) end-flowering stage. The contents of total phenolics, flavanoids, individual phenolic compounds as well as antioxidant and tyrosinase inhibitory activity of peach blossoms at different developmental stages were investigated. The total phenolic contents varied from 149.80 to 74.80 mg chlorogenic acid equivalents/g dry weight (DW), and the total flavanoid contents ranged from 93.03 to 44.06 mg rutin equivalents/g DW. Both the contents of total phenolics and flavanoids decreased during blossom development. Chlorogenic acid was the predominant component, accounting for 62.08%–71.09% of the total amount of identified phenolic compounds in peach blossom. The antioxidant capacities determined by different assays and tyrosinase inhibitory activity also showed descending patterns during blossom development. Significant correlations were observed between antioxidant capacities with contents of total phenolics and total flavanoids as well as chlorogenic acid, cinnamic acid and kaempferol-3-*O*-galactoside, while the tyrosinase inhibitory activity had lower correlations with total phenolics and total flavanoids as well as chlorogenic acid, quercetin-3-*O*-rhamnoside, kaempferol-3-*O*-galactoside and cinnamic acid. The antioxidant activities of peach blossom seemed to be more dependent on the phenolic compounds than tyrosinase inhibitory activity.

## 1. Introduction

Free radicals and other reactive oxygen species are intermediate metabolites in many biochemical reactions of living organisms with high reactivity. They can react with cellular components such as lipids, proteins, polypeptides, nucleic acids, saccharides and organic acids, and thereby cause cell damage [1,2,3]. Oxidative damage has been associated with the development of various diseases and degenerative processes in ageing [4,5], and consumption of foods or plant extracts rich in antioxidant phytochemicals is believed to be able to reduce the deleterious effects of oxidative stress [5,6,7,8]. Among the sources of these natural antioxidants, fruits, vegetables and herbs are well documented due to their high contents of ascorbic acid, phenolics, carotenoids and potent antioxidant capacity [9,10,11].

Tyrosinase (EC1.14.18.1) is a key enzyme in melanin biosynthesis. It catalyzes the hydroxylation of monophenols to *o*-diphenols (monophenolase activity) and the subsequent oxidation of *o*-diphenols to corresponding *o*-quinones (diphenolase activity) [12]. These *o*-quinones then polymerize spontaneously to form brown pigments of high molecular weight (melanins), which is responsible for pigmentation and the color patterns of mammalian skin and hair [13,14]. However, excessive accumulation of epidermal pigmentation may cause several dermatological diseases or hyperpigmentation disorders, such as melanomas, melasma, freckles and age spots [14]. Furthermore, tyrosinase is also responsible for the enzymatic browning of plant tissue, resulting in unfavorable color change and decrease in nutritional value of plant-derived foods [15,16]. Therefore, tyrosinase inhibitors may play an important role in cosmetic and medicinal products as well as agricultural and food industry [17,18,19]. A large number of tyrosinase inhibitors from natural sources have been identified and characterized, in which the phenolic compounds are the largest groups [17,18,19,20,21]. Furthermore, many plant materials, such as herbs, gingers, pomegranate peel, mango seed kernel, green tea seed, oriental cherry, mulberry twigs and root bark have been investigated for their tyrosinase inhibitory activity and several studies have ascribed it to their phenolic compounds [22,23,24,25,26,27,28,29].

Peach (*Prunus persica*) originated in China and is now cultivated all over the world. The peach fruit is nutritious and rich in antioxidant phytochemicals [30,31]. The peach blossoms have long been used in traditional Chinese medicine to lighten skin, dispel blood stasis and eliminate facial melasma, freckles and age spots. Usually, the dried peach blossoms are consumed as tea or wine and believed to be beneficial for human health. Previous studies revealed that the peach blossom extracts rich in phenolic compounds have potent antoxidant and tyrosinase inhibitory activities [32,33,34]. However, biosynthesis of these bioactive phytochemicals in plant tissues is a highly ordered process and varies during the developmental periods, and the substantial differences in biological activities may occur as well [35,36]. Therefore, determination of the contents of bioactive phytochemicals as well as corresponding bioactivities in plant tissues at different developmental stages is essential for optimizing harvesting time to obtain maximum natural production with higher bioactivities. The present work aims to investigate the changes of phenolics, flavanoids, antioxidant and tyrosinase inhibitory activities of peach blossoms at different development stages. Since different antioxidants may act through different mechanisms, and the mechanism involved in a particular test system depends on the composition, pH and redox potentials of the system [37,38], no single method for antioxidant evaluation can provide full understanding about the antioxidant effects of foods and other materials. For this reason, various methods for antioxidant evaluation, including Photochem assay, DPPH (1,1-diphenyl-2-picrylhydrazyl) assay and hydroxyl radical assay were used in this study.

## 2. Results and Discussion

### 2.1. Total Phenolics and Flavanoids

The freshly-harvested peach blossoms were classified into six groups according to their botanical characteristics [39]: (I) bud emerging stage; (II) middle bud stage; (III) large bud stage; (IV) initial-flowering stage; (V) full-flowering stage; and (VI) end-flowering stage. After removing receptacles, sepals, pistils and stamens, the petals were dried in the shade at room temperature until ready for use.

**Figure 1:**
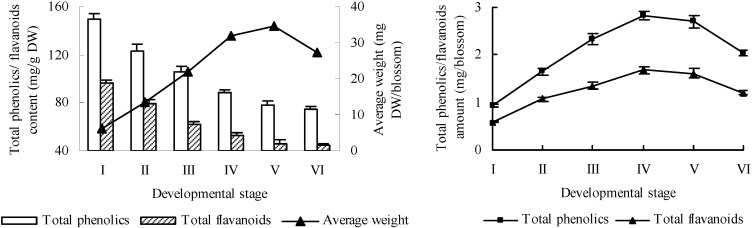
Total phenolic and flavanoid contents of peach blossoms at different developmental stages.

The total phenolic and flavanoid contents of peach blossoms at different developmental stages were measured by using a Folin–Ciocalteu assay [40] and NaNO_2_–Al(NO_3_)_3_–NaOH test system [41], respectively. As shown in Figure 1, the total phenolic and flavanoid contents of peach blossoms declined during development. Especially at bud emerging stage (I) to initial-flowering stage (IV), the contents of total phenolics and flavanoids decreased dramatically from 149.80 and 88.13 mg/g dry weight (DW) to 96.03 and 52.40 mg/g DW, respectively. After initial-flowering stage (IV), the decreasing tendency alleviated, and the total phenolic and flavanoid contents of peach blossoms at full-flowering stage (V) and end-flowering stage (VI) had no significant difference. Meanwhile, the average weight per blossom increased quickly before initial-flowering stage (IV), while the blossoms of full-flowering stage (V) only showed a slightly higher average weight than that of initial-flowering stage (IV). Furthermore, the average weight of blossoms at end-flowering stage (VI) showed a dramatic decrease as compared with that of full-flowering stage (V). Due to the change of average weight of peach blossoms, the amounts of total phenolics and flavanoids calculated as mg/blossom showed an increase pattern during development from bud emerging stage (I) to initial-flowering stage (IV), and then declined at full-flowering stage (V) and end-flowering stage (VI). The total amounts of phenolics and flavanoids of blossoms at initial-flowering stage (IV) were increased by 2.05 and 1.83 fold compared to those of bud emerging stage (I). Therefore, the initial-flowering stage (IV) might be the optimal harvesting time to obtain maximum phenolics and flavanoids production for peach blossom.

Many researchers have observed the decrease of total phenolic and/or flavanoid contents of other plant tissues during development [42,43,44,45]. These might mainly be due to the rapid growth of plant tissues in the early period, leading to a dilution effect of phenolic compounds. In the late period, the plant tissue grows slowly, while the oxidation and transformation of phenolic compounds may be accelerated due to the senescence of plant tissue. In our previous research, we had reported a negative correlation between polyphenols content and fruit weight during apple fruit development, suggesting that the increase of fruit weight in early period may be one of the main reasons of the decline of phenolic content during fruit development [45]. Wei *et al.* [46] also found the decline of some active components in leaves of pigeon pea during vegetative growth and ascribed it to the rapid growth of plant during that period.

### 2.2. High Performance Liquid Chromatography (HPLC) Analysis of Selected Phenolic Compounds

**Figure 2:**
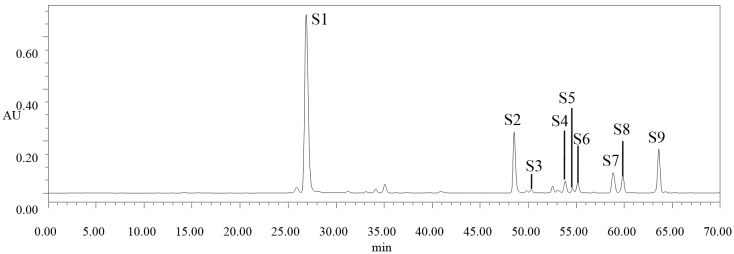
Typical HPLC chromatogram of peach blossom extract at 280 nm. *S1*: chlorogenic acid; *S2*: unknown; *S3*: quercetin-3-*O*-rhamnoside; *S4*: kaempferol-3-*O*-galactoside; *S5*: quercetin-3-*O*-galactoside; *S6*: kaempferol-4-*O*-glucoside; *S7*: unknown; *S8*: cinnamic acid; and *S9*: unknown.

Phenolic composition of peach blossom extract was analyzed by using a 1525 HPLC system coupled with a 2996 Photodiode Array Detector (Waters Corp., Wilford, MA, USA). By comparing the retention time and UV spectra with those of phenolic standards, chlorogenic acid (*S1*), quercetin-3-*O*-rhamnoside (*S3*), kaempferol-3-*O*-galactoside (*S4*), quercetin-3-*O*-galactoside (*S5*), kaempferol-4-*O*-glucoside (*S6*), and cinnamic acid (*S8*) were identified from the peach blossom extract (Figure 2). Several “unknown” compounds were also presented in the chromatogram. For example, the compound noted as “*S2*” had similar UV spectra to chlorogenic acid, and “*S7*” and “*S9*” had similar UV spectra to kaempferol-4-*O*-glucoside. However, they were not confirmed due to the absence of corresponding standards with the same retention time and UV spectra. Therefore, some other analytical technologies or phenolic standards are needed for elucidating the phenolic composition of peach blossom extract in further studies. Among them, chlorogenic acid was the predominant component in peach blossom extract, which accounted for 62.08%–71.09% of the total amount of identified phenolic compounds (Table 1). During blossom development, the content of chlorogenic acid decreased continuously with a variation of 57.92% from bud emerging stage (I) to end-flowering stage (VI), which was consistent with the change of total phenolic and flavanoid contents. The content of cinnamic acid also showed a similar change during blossom development. For quercetin-3-*O*-rhamnoside, kaempferol-3-*O*-galactoside, quercetin-3-*O*-galactoside and kaempferol-4-*O*-glucoside, only small differences were observed among different developmental stages of blossoms, and the changes were diverse for each compound during blossom development. For example, the content of quercetin-3-*O*-rhamnoside showed an obvious decrease till after initial-flowering stage (IV), while the content of kaempferol-4-*O*-glucoside increased significantly at end-flowering stage (VI). Furthermore, no significant change was found in content of quercetin-3-*O*-galactoside during the blossom development.

**Table 1:** Contents of individual phenolic compounds in peach blossoms at different developmental stages (mg/g dry weight).

Developmental Stage	I	II	III	IV	V	VI
Chlorogenic acid	72.87 ± 3.41 ^a^	59.58 ± 3.67 ^b^	44.21 ± 1.79 ^c^	37.32 ± 2.30 ^d^	32.53 ± 2.85 ^de^	30.67 ± 3.13 ^e^
Quercetin-3-*O*-rhamnoside	3.72 ± 0.30 ^ab^	4.00 ± 0.18 ^a^	4.00 ± 0.26 ^a^	3.76 ± 0.14 ^ab^	3.36 ± 0.26 ^bc^	3.07 ± 0.21 ^c^
Kaempferol-3-*O*-galactoside	9.96 ± 0.43 ^a^	10.08 ± 0.52 ^a^	8.45 ± 0.35 ^b^	7.50 ± 0.46 ^c^	6.52 ± 0.29 ^d^	7.05 ± 0.23 ^cd^
Quercetin-3-*O*-galactoside	0.80 ± 0.05 ^a^	0.89 ± 0.05 ^a^	0.86 ± 0.02 ^a^	0.83 ± 0.04 ^a^	0.81 ± 0.03 ^a^	0.84 ± 0.03 ^a^
Kaempferol-4-*O*-glucoside	5.57 ± 0.22 ^b^	5.86 ± 0.19 ^b^	5.62 ± 0.31 ^b^	5.61 ± 0.28 ^b^	5.99 ± 0.17 ^b^	6.96 ± 0.33 ^a^
Cinnamic acid	1.48 ± 0.10 ^a^	1.39 ± 0.06 ^a^	1.13 ± 0.03 ^b^	0.98 ± 0.03 ^b^	0.82 ± 0.05 ^c^	0.82 ± 0.02 ^c^

Different superscripts between rows represent significant differences between samples (*p* < 0.05).

### 2.3. Antioxidant Activity

#### 2.3.1. Scavenging Capacity towards Superoxide Anion Radicals

**Figure 3:**
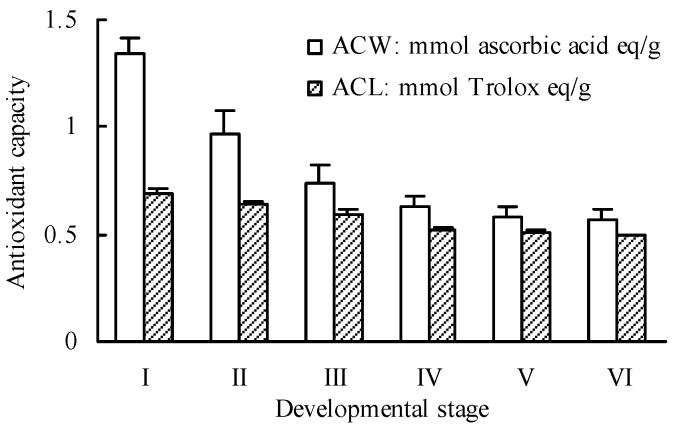
Scavenging effects towards superoxide anion radicals of peach blossoms at different developmental stages. ACW: Antioxidative Capacity in Water-soluble substances; ACL: Antioxidative Capacity in Lipid-soluble substances.

The superoxide anion radical scavenging capacities of peach blossoms at different developmental stages were evaluated using a photochemiluminescence assay that was conducted with the PHOTOCHEM^®^ device (Analytik Jena AG, Jena, Germany) [47]. As shown in Figure 3, all of the peach blossoms at different developmental stages exhibited strong scavenging capacities towards superoxide anion radicals in both ACW (Antioxidative Capacity in Water-soluble substances) and ACL (Antioxidative Capacity in Lipid-soluble substances) systems. During blossom development from bud emerging stage (I) to end-flowering stage (VI), the superoxide anion radical scavenging capacity showed a descending pattern with a decrease of 57.52% for water-soluble antioxidant capacity and 28.13% for lipid-soluble antioxidant capacity. Since the two different protocols of ACW and ACL were used to evaluated the antioxidant capacity of the water-soluble and lipid-soluble components, respectively [47,48], the difference in changes of ACW and ACL value indicated that the content of total water-soluble antioxidant components in peach blossoms decreased more greatly than that of lipid-soluble antioxidant components during development.

Besco *et al.* [48] investigated the antioxidant capacities of baobab and several fruits rich in vitamin C using the same method as the present research. The ACW value of baobab, kiwi, orange, bilberry, and strawberry were 1.2–386.0 (depending on the plant part), 0.73, 17.0, 1.95, and 1.72 μmol AA/g, and the ACL value were 1.0–508.0 (depending on the plant part), 0.27, 0.29, 2.00, and 0.36, respectively. Even if the relative water contents of samples are considered, the ACW and ACL values of peach blossoms are still much higher than the above results. This indicates that the peach blossoms have a potent antioxidant activity and may play an important role in preventing human health from oxidative press.

#### 2.3.2. Scavenging Capacity towards Hydroxyl Radicals

**Figure 4:**
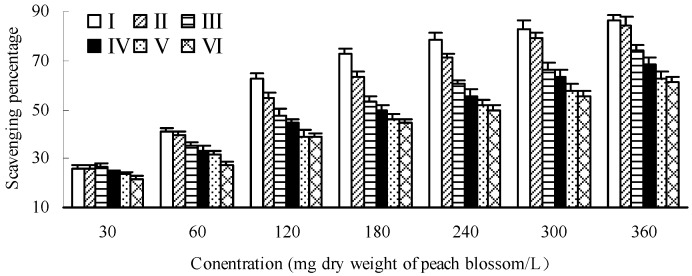
Scavenging effects towards hydroxyl radicals of peach blossoms at different developmental stages.

As shown in Figure 4, the hydroxyl radical scavenging effects showed a dose-dependent pattern for each developmental stage of peach blossoms. There were no significant differences among different developmental stages at lower sample concentration, but the differences became greater with the increase of concentration. At concentration of 240 mg/L, the scavenging percentage of blossoms at bud emerging stage (I) was higher by 57.90% than that of end-flowering stage (VI), while it was only 19.80% at concentration of 30 mg/L. On the whole, the scavenging capacity towards hydroxyl radicals of peach blossoms decreased during the development of blossoms. The blossom of bud emerging stage (I) had the strongest scavenging capacity towards hydroxyl radicals with an IC_50_ of 85.41 mg/L, and that of end-flowering stage (VI) was the poorest with an IC_50_ of 243.84 mg/L.

#### 2.3.3. Scavenging Capacity towards DPPH Radicals

**Figure 5:**
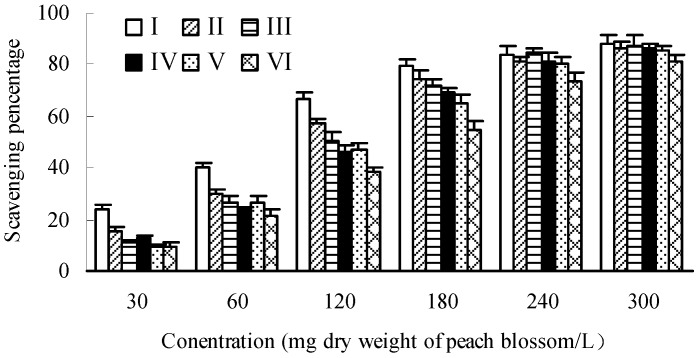
Scavenging effects towards DPPH (1,1-diphenyl-2-picrylhydrazyl) radicals of peach blossoms at different developmental stages.

Differing from hydroxyl radical assay, the differences of DPPH (1,1-diphenyl-2-picrylhydrazyl) radical scavenging capacities among different developmental stages at lower sample concentration were more significant than those of higher sample concentration (Figure 5). At concentration of 30 mg/L, the scavenging percentage of blossoms at bud emerging stage (I) was higher by 148.62% than that of end-flowering stage (VI), while it was only 13.37% at concentration of 240 mg/L. Moreover, the DPPH radical scavenging effects also showed a dose-dependent pattern and decreased during the development of blossoms, with a variation of IC_50_ from 82.00 mg/L at bud emerging stage (I) to 162.16 mg/L at end-flowering stage (VI).

As compared with hydroxyl radical assay, the scavenging capacity towards DPPH radicals of peach blossoms was stronger. Zhang *et al.* [49] determined the antioxidant capacity of pomegranate flower petal and obtained an IC_50_ of 344.35 and 107.78 mg/L for DPPH and hydroxyl radical assay, respectively, indicating that the pomegranate flower petal extract could scavenging hydroxyl radical more efficiently. This difference may be ascribed to the difference of composition of test samples, and thereby the reaction process as well as the mechanism involved may also differ from each other.

### 2.4. Tyrosinase Inhibitory Activity

**Figure 6:**
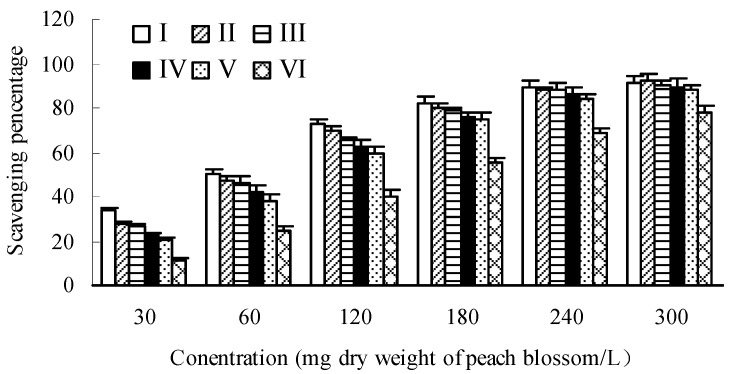
Tyrosinase inhibitory effects of peach blossoms at different developmental stages.

Tyrosinase inhibitory activity of peach blossom was also influenced by developmental period. As shown in Figure 6, the inhibitory effects varied among different developmental stages at each concentration, showing a descending tendency with the development of blossoms. Especially at the end period, a sharp decrease was observed from full-flowering stage (V) to end-flowering stage (VI). Furthermore, the tyrosinase inhibitory activity of peach blossom was also dependent on concentration, and the IC_50_ for bud emerging stage (I) to end-flowering stage (VI) were 59.52, 67.08, 71.96 82.68, 92.44, and 158.68 mg/L, respectively.

Many flowers have been investigated for their tyrosinase inhibitory activity. For example, Jo *et al.* [50] reported the tyrosinase inhibitory activity of magnolia flower extract with an IC_50_ of 3343.58 mg/L and Vallisuta *et al.* [51] demonstrated that marigold flower ethanol extract possessed tyrosinase inhibitory activity with an IC_50_ of 1078 mg/L. Mango seed kernel and tea seed pericarp extract were also reported for their tyrosinase inhibitory activity with an IC_50_ of 98.63 and 735.58 mg/L, respectively [27,52]. The present research suggests that peach blossom extract be a potent tyrosinase inhibitor and of interest for cosmetic development.

### 2.5. Correlation Analysis

Based on ACW, ACL, or IC_50_ value, correlation analysis was performed between the total phenolic, flavanoid, individual phenolic contents and antioxidant, tyrosinase inhibitory activity of peach blossoms at different developmental stages. As shown in Table 2, significant correlations were observed between antioxidant capacities determined by different assays with contents of total phenolics and total flavanoids as well as chlorogenic acid, cinnamic acid and kaempferol-3-*O*-galactoside (*p <* 0.01). Many studies have documented the correlation between phenolics and antioxidant capacity [53,54,55,56,57], the antioxidant activity of chlorogenic acid, cinnamic acid, kaempferol derivates and quercetin derivates were also reported [58,59,60]. The results of present research were partly in agreement with the previous results. However, quercetin-3-*O*-rhamnoside, quercetin-3-*O*-galactoside, and kaempferol-4-*O*-glucoside, did not show a good correlation with the antioxidant activities in this research. This may mainly be due to their low contents in the peach blossoms as compared to chlorogenic acid.

The tyrosinase inhibitory activity of peach blossom also showed positive correlations with the contents of total phenolics and total flavanoids as well as chlorogenic acid, quercetin-3-*O*-rhamnoside, kaempferol-3-*O*-galactoside and cinnamic acid (*r* = 0.6995~0.8648). However, the correlations were relatively lower as compared to those of antioxidant activities. Other researchers also reported the low positive correlation of phenolics and flavonoids with tyrosinase inhibitory activity [61,62]. This indicates that the antioxidant activities of peach blossom may be more dependent on the phenolic compounds than tyrosinase inhibitory activity.

Interestingly, the tyrosinase inhibitory activity was significantly correlated with DPPH scavenging capacity (*p <* 0.01) and hydroxyl radical scavenging capacity (*p <* 0.05). Masuda *et al.* [63] had reported that seashore plant species with strong antioxidant capacity also had strong tyrosinase inhibition activity, which was similar to our results. It was suggested that the antioxidant activity might be one of the important mechanisms for tyrosinase inhibitory activity.

**Table 2:** Correlations between total phenolic, total flavanoid, and individual phenolic contents and antioxidant and tyrosinase inhibitory activity.

	ACW	ACL	HR	DPPH	TI
Total phenolics	0.9795 **	0.9875 **	0.9593 **	0.9372 **	0.7242
Total flavanoids	0.9775 **	0.9845 **	0.9608 **	0.9302 **	0.7124
Chlorogenic acid	0.9856 **	0.9795 **	0.9496 **	0.9285 **	0.6995
Quercetin-3-*O*-rhamnoside	0.4525	0.6602	0.8037 *	0.7063	0.8648 *
Kaempferol-3-*O*-galactoside	0.8825 **	0.9510 **	0.9585 **	0.8369 *	0.6570
Quercetin-3-*O*-galactoside	−0.1236	0.1176	0.2104	−0.0640	0.0501
Kaempferol-4-*O*-glucoside	−0.4788	−0.5769	−0.7043	−0.7862 *	−0.9560 **
Cinnamic acid	0.9333 **	0.9802 **	0.9851 **	0.9136 **	0.7379
TI	0.6282	0.7427	0.8368 *	0.9001 **	
DPPH	0.9011 **	0.9355 **	0.9435 **		
HR	0.8899 **	0.9652 **			
ACL	0.9509 **				

ACW: antioxidant capacity in water-soluble substances; ACL: antioxidant capacity in lipid-soluble substances; HR: hydroxyl radical scavenging capacity. DPPH: DPPH radical scavenging capacity; TI: tyrosinase inhibitory activity. * indicated significant correlation at *p* *<* 0.05, ** indicated significant correlation at *p* *<* 0.01.

## 3. Experimental Section

### 3.1. Materials

The peach blossoms were harvested from trees grown in National Fruit Tree Germplasm Repository in Zhengzhou Fruit Research Institute, Chinese Academy of Agricultural Sciences, China. The freshly-harvested blossoms were classified into six groups according to their botanical characteristics [39]: (I) bud emerging stage: buds initially emerged from scaly bracts, a small part of petals displayed from the top of buds; (II) middle bud stage: buds swelled constantly, half petals of the bud displayed; (III) large bud stage: the displayed petals accounted for 80 percent of the bud; (IV) initial-flowering stage: the outer petals began to open; (V) full-flowering stage: petals opened completely with observable pistils and stamens; and (VI) end-flowering stage: fully matured petals began to fall. Then, the receptacles, sepals, pistils and stamens were removed and the petals were dried in the shade at room temperature.

### 3.2. Preparation of Peach Blossom Extracts

The dried petals of peach blossoms were ground to fine powder to pass through a 60-mesh sieve. In total, 0.5 g of the powder was extracted with 50 mL of 50% ethanol at 25 °C for 6 h. Then, the extracts were filtered to yield ethanolic extracts of peach blossoms. These ethanolic extracts were used for the determination of total phenolics, flavanoids, antioxidant and tyrosinase inhibitory activity.

### 3.3. Determination of Total Phenolic Content

Folin–Ciocalteu reagent was used to determine the total phenolic content according to the method described by Singleton *et al.* [40]. The absorbance was measured at 760 nm with a UV/Vis Spectrometer and chlorogenic acid was used as standard phenolic compound for the calibration curve. Total phenolic contents of peach blossoms were expressed as milligram of chlorogenic acid equivalents (CAE) per gram dry weight of peach blossoms (mg CAE/g DW).

### 3.4. Determination of Total Flavonoid Content

A colorimetric method with NaNO_2_–Al(NO_3_)_3_–NaOH test system was used to estimate the total flavonoid content as described by Yang *et al.* [41]. The absorbance was measured at 510 nm with a UV/Vis Spectrometer and rutin was used as standard flavonoid compound for the calibration curve. Total flavonoid contents of peach blossoms were expressed as milligram of rutin equivalents (RE) per gram dry weight of peach blossoms (mg RE/g DW).

### 3.5. High Performance Liquid Chromatography (HPLC) Analysis of Selected Phenolic Compounds

Phenolic composition of peach blossom extract was analyzed by using a 1525 HPLC system coupled with a 2996 Photodiode Array Detector (Waters Corp., Wilford, MA, USA). The separation was performed on a Waters Symmetry C18 column (4.6 × 150 mm, 5 µm) at 30 °C with a gradient elution of mobile phase consisting of methanol (solvent A) and deionized water of pH 2.6 adjusted with phosphoric acid (solvent B). The flow rate of solvent was 0.6 mL/min, and the gradient elution program was as follows: 0 min 15% A, 15 min 25% A, 25 min 25% A, 65 min 75% A, 70 min 15% A. Peaks were monitored at 280 nm with a scanning range from 210 to 400 nm, and the phenolic compounds in peach blossom extract were identified by comparing the retention time and UV spectra with those of phenolic standards. Quantitative analysis of identified phenolic compounds was conducted by using external calibration curves, and the contents of the individual phenolic compounds were expressed as milligram per gram dry weight of peach blossoms (mg/g DW).

### 3.6. Photochem Assay

A photochemiluminescence assay was conducted with the PHOTOCHEM^®^ device (Analytik Jena AG, Jena, Germany) as described by Pegg *et al.* [47]. Superoxide anion radicals were generated by optical excitation of luminol (5-amino-2,3-dihydro-l,4-phthalazinedione), and the water-soluble and lipid-soluble antioxidant capacity of peach blossom extract was measured by using ACW (Antioxidative Capacity in Water-soluble substances) and ACL (Antioxidative Capacity in Lipid-soluble substances) kits, respectively. Results were calculated through the ascorbic acid calibration curve for water-soluble antioxidant capacity or Trolox (a synthetic vitamin E) calibration curve for lipid-soluble antioxidant capacity and expressed as mmol equivalents per gram dry weight of peach blossoms (mmol ascorbic acid eq/g DW or mmol Trolox eq/g DW).

### 3.7. DPPH Assay

Scavenging capacity towards DPPH (1,1-diphenyl-2-picrylhydrazyl) radicals was assayed according to the method described by Maisuthisakul and Gordon [29] with slight modifications. Briefly, 1.0 mL of 0.2 mmol/L of DPPH ethanol solution was added to 2.0 mL of peach blossom extracts at various concentrations or negative control (1:1 ethanol–water). The mixture was shaken vigorously and incubated at 25 °C in the dark for 30 min. Then the absorbance was measured at 517 nm with a spectrophotometer. Lower absorbance of the reaction mixture indicates higher free radical scavenging activity. The DPPH radical scavenging capacity (%) was calculated by using the following equation:

Scavenging percentage = [A_0_ – (A_1_ – A_2_) ] × 100/A_0_ (1)

where A_0_ is the absorbance of the negative control without peach blossom extract, A_1_ is the absorbance in the presence of peach blossom extract and DPPH, and A_2_ is the absorbance of peach blossom extract without DPPH.

### 3.8. Hydroxyl Radical Assay

Scavenging capacity towards hydroxyl radicals was measured according to the method described by Smirnoff and Cumbes [64] with slight modifications. The reaction mixture contained 0.5 mL of 5.5 mmol/L FeSO_4_, 0.5 mL of 6.5 mmol/L EDTA, 0.5 mL of 10 mmol/L sodium salicylate, 0.5 mL of 12 mmol/L hydrogen peroxide, 3.0 mL of 50 mmol/L phosphate buffer (pH 7.4) and 0.5 mL of samples. After incubation for 30 min at 37 °C, the absorbance of the mixture was measured at 510 nm with a spectrophotometer. The scavenging percentage was calculated as follows:

Scavenging percentage = [A_0_ – (A_1_ – A_2_)] × 100/A_0_ (2)

where A_0_ is the absorbance of control without peach blossom extract, A_1_ is the absorbance in the presence of peach blossom extract and sodium salicylate, and A_2_ is the absorbance of peach blossom extract without sodium salicylate.

### 3.9. Tyrosinase Inhibition Assay

Tyrosinase inhibitory activity was determined by using a commercially available mushroom tyrosinase with *L*-DOPA (*L*-3,4-dihydroxyphenylalanine) as substrate according to the method described by Hsu *et al.* [65] with slight modifications. Each 4 mL of reaction mixture contained 1.5 mmol/L *L*-DOPA, 21 U of mushroom tyrosinase, 50 mmol/L phosphate buffer (pH 6.8), and peach blossom extracts at various concentrations. The reaction was performed at 30 °C and monitored by measuring the absorbance at 475 nm for 3 min. The tyrosinase inhibitory activity was calculated according to the following equation:

Tyrosinase inhibition percentage = [(*A* – *B*) – (*C* – *D*)] × 100/(*A* – *B*) (3)

where A is the absorbance of the control in the presence of *L*-DOPA and tyrosinase, B is the absorbance of *L*-DOPA, C is the absorbance of the sample in the presence of peach blossom extract, *L*-DOPA and tyrosinase, and D is the absorbance of sample control in the presence of peach blossom extract and *L*-DOPA. 

### 3.10. Statistical Analysis

Statistical analysis was performed with IBM SPSS Statistics 19 (IBM-SPSS Inc, Armonk, NY, USA). The data are presented as mean ± SD for triplicate.

## 4. Conclusions

The total phenolic and flavanoid contents of peach blossoms declined during development with a variation from 149.80 mg CAE/g DW and 88.13 mg RE/g DW to 74.80 mg CAE/g DW and 44.06 mg RE/g DW, respectively. Meanwhile, the average weight per blossom increased quickly before initial-flowering stage (IV), leading to an increase of total amounts of phenolics and flavanoids per blossom up to initial-flowering stage (IV). Thus, the initial-flowering stage (IV) might be the optimal harvesting time to obtain maximum phenolics and flavanoids production for peach blossom.

Six phenolic compounds were identified and quantified, and chlorogenic acid was the predominant component in peach blossom extract, accounting for 62.08%–71.09% of the total amount of identified phenolic compounds. It decreased continuously with a variation of 57.92% from bud emerging stage (I) to end-flowering stage (VI), which was consistent with the change of total phenolic and flavanoid contents. The content of cinnamic acid also showed a similar change during blossom development. However, for quercetin-3-*O*-rhamnoside, kaempferol-3-*O*-galactoside, quercetin-3-*O*-galactoside and kaempferol-4-*O*-glucoside, only small differences were observed among different developmental stages of blossoms, and the changes were diverse for each compound during blossom development.

The antioxidant capacities determined by different assays and tyrosinase inhibitory activity also showed descending patterns during blossom development. Significant correlations were observed between antioxidant capacities determined by different assays with contents of total phenolics and total flavanoids as well as chlorogenic acid, cinnamic acid and kaempferol-3-*O*-galactoside, while the tyrosinase inhibitory activity of peach blossom had lower correlations with total phenolics and total flavanoids as well as chlorogenic acid, quercetin-3-*O*-rhamnoside, kaempferol-3-*O*-galactoside and cinnamic acid. The antioxidant activities of peach blossom seemed to be more dependent on the phenolic compounds than tyrosinase inhibitory activity.
